# Controllable Preparation of Oriented Boron Nitride Nanosheets/Polyacrylate Pressure-Sensitive Adhesive Composites with Enhanced Thermal Conductivity

**DOI:** 10.3390/polym17121604

**Published:** 2025-06-09

**Authors:** Yuan Liu, Chaochao Cao, De Zheng, Guohua Li, Xiongwei Qu

**Affiliations:** 1 Hebei Key Laboratory of Functional Polymers, School of Chemical Engineering, Hebei University of Technology, Tianjin 300130, China; ly9901232025@163.com (Y.L.); nkligh@126.com (G.L.); 2 Guangdong Winner New Material Technology Co., Ltd., Foushan 528524, China; gmwinner@vip.163.com

**Keywords:** boron nitride nanosheets, bulk polymerization, thermal conductivity, orientation, pressure sensitive adhesive

## Abstract

Traditional approaches to constructing thermally conductive networks typically necessitate costly equipment and intricate processes, rendering them unsuitable for mass production and commercialization. Here, we propose a facile strategy to construct highly oriented boron nitride/polyacrylate pressure-sensitive adhesive frameworks by a calendering process. A UV light-based bulk polymerization method is adopted to prepare the pressure-sensitive adhesives (PSAs), which makes the preparation process solvent-free and volatile organic compound (VOC)-free, and environmentally friendly compared to emulsion and solvent-based pressure-sensitive adhesives. This simple, economical and scalable method provides new ideas and ways for the preparation of advanced thermal conductive networks. The highly oriented and flexible m-BNNSs/polyacrylate pressure-sensitive adhesive composites (m-BNNSs/PSAs-Ori) exhibited a significantly high thermal conductivity (TC) of 0.9552 W/(m·K) at 25 wt% filler content. Significantly, m-BNNSs/PSAs-Ori composites showed a better thermal response than the single-layer thermally conductive pressure-sensitive adhesive. Moreover, the composites also possess excellent electrical insulation and mechanical properties. This exploration not only provides a reasonable design scheme for thermal interface materials, but also promotes the practical application of polyacrylate pressure-sensitive adhesive composites in thermal management.

## 1. Introduction

With the miniaturization of electronic devices and the increasing power density, thermal management materials with high thermal conductivity have received much attention [1,2,3]. Among various thermal management strategies, the design and fabrication of thermal interface materials (TIMs) with excellent thermal conductivity and electrical insulation is considered to be an effective strategy for maintaining reliability and extending the service life of electronic devices [4,5,6,7,8,9]. High-performance TIMs exhibiting exceptional flexibility and elevated TC, particularly efficient heat dissipation along the entire thickness direction, are crucial for effective thermal management in next-generation soft electronics and robotics [10,11,12]. Acrylate-based PSA has been widely used in the manufacture of automotive components to electronics due to its excellent flexibility, light weight, low cost and processability [13,14].

Acrylate-based pressure-sensitive adhesives are widely used because of their simple manufacturing process, short production cycle, excellent weathering resistance, low cost and colorless and transparent coating film. There are four types of acrylate PSAs on the market: solvent-based [15], emulsion-based [16], hot-melt [17] and radiation-cured [18]. In contrast, UV-cured PSAs offer undisputed advantages over the other types because they contain no organic solvents and have fast curing speeds and good performance. However, low TC limits their use as thermal interface materials. Polymer matrix composites with high out-of-plane TC are needed to fulfill the heat dissipation requirements [19,20,21,22]. Substantial research efforts have been dedicated to the development of polymer-based nanocomposites.

Hexagonal boron nitride nanosheets (BNNSs) have significant anisotropic thermal conductivity, which is much higher in the in-plane direction (~400 W/(m·K) than in the out-of-plane direction (~2 W/(m·K). Polymer/boron nitride (BN) composites with high in-plane TC (300 W/(m·K)) and excellent insulating properties are a promising TIM [23,24]. Utilizing the anisotropic characteristics of lamellar BN, aligning the orientation of lamellar BN within the matrix serves as an effective approach to enhance the TC of composites. This alignment can be achieved through various methods, including two-roll milling, hot pressing, rod coating, 3D printing, electric or magnetic field induction, electrostatic spinning, as well as the ice template technique [25,26,27,28,29,30,31,32,33]. For example, Yuan et al. used a magnetic field to induce the orientation of BN in polymers and found that the TC of the oriented composites increased by 44.5% with the addition of a filler volume fraction of 9.14% [34]. Yin et al. prepared silicone rubber (MVQ)/oriented boron nitride (ABN) composites using a roll-cutting method. The prepared oriented composites showed more excellent TC compared to the unoriented pressure-sensitive adhesive [35]. Zhong et al. synthesized BN nanosheets (BNNSs)/SR composites incorporating oriented BNNSs via double-roller milling [36]. The resultant composites exhibited a 22% enhancement in TC in comparison to those with stochastically dispersed BNNS. Nevertheless, the TC acquired in this manner is not sufficiently high, likely due to the inadequate degree of orientation.

Therefore, the researchers integrated a range of integrated strategies aimed at enhancing the orientation degree of BN, such as firstly using electrostatic spinning and shearing to realize the preorientation of BN, followed by hot pressing or multilayer hot pressing to additionally improve the degree of orientation. Liu et al. used a multilayer stacked hot pressing method to realize the preparation of high TC silicone rubber/boron nitride composites, and based on the test characterization, the orientation of BN reinforcement and the excellent heat transfer properties of the composites were confirmed, and the TC of the composites reached 22.08 W/(m·K) [37]. Guo et al. used a gradient temperature field to fabricate the alternating layers of GO and BN by ice template method, and the polyimide composites with 1.49 W/(m·K) in-plane TC were realized under the filler loading of 3.8 wt% at the temperature of 3.8 wt%. Polyimide composites with an in-plane thermal conductivity of 1.49 W/(m·K) were realized at 3.8 wt% filler loading [38]. Song et al. prepared highly oriented cellulose/BNNS composites using strain-induced and post-compression strategies, with a thermal conductivity of 40.23 W/(m·K) at a BN content of 20 wt% [39]. As a result, the current methodologies for attaining BN orientation exhibit both inconvenience and incompatibility with industrial-scale production. Consequently, there exists an urgent demand for innovative and facile preparation techniques to fabricate high-performance thermal interface materials.

In this work, we used an environmentally friendly and efficient UV bulk polymerization method to prepare polyacrylate pressure-sensitive adhesives with controllable thickness, and realized the orientation of m-BNNS fillers in pressure-sensitive adhesives through a simple calendering process. When the filler content was 25 wt%, the TC of the composites reached 0.9552 W/(m·K), which is 543% higher than that of pure pressure-sensitive adhesives, after five layers of coating orientation. It shows that the m-BNNSs have a higher degree of selective orientation, a better TC path and a lower interfacial thermal resistance, which was verified by means of SEM, XRD and infrared thermography. The prepared composites exhibit excellent electrical insulation properties, thermal stability and mechanical properties. The proposed multilayer coating and calendering process has a promising future for large-scale and continuous production, and the prepared composites have broad application prospects in fields such as electronic devices.

## 2. Experimental Sections

### 2.1. Materials

Hexagonal boron nitride (h-BN), Shandong Qingzhou Fangyuan Boron Nitride Factory, Weifang, China; diphenyl (2,4,6-trimethylbenzoyl)-phosphine oxide (TPO), Tianjin Jiuri Chemistry Co.,Tianjin, China; 1,6-hexanediol diacrylate (HDDA), Shanghai McLean Biochemical Technology Co., Shanghai, China; butyl acrylate (BA), Shanghai Aladdin Biochemical Technology Co., Ltd., Shanghai, China; hydroxyethyl acrylate (HEA), Shanghai Bider Pharmaceutical Technology Co., Ltd., Shanghai, China; and acrylic acid (AA), Tianjin Kemiou Chemical Reagent Co., Tianjin, China were purchased and used without further purified. Isopropyl alcohol (IPA), Tianjin Kemiou Chemical Reagent Co., Tianjin, China; γ-(2, 3-epoxy-propoxy) propyl trimethoxysilane (KH560), Shanghai Bider Medical Technology Co., Ltd, Shanghai, China.

### 2.2. Preparation of m-BNNSs

The preparation of m-BNNSs can be divided into two main steps, namely liquid-phase ultrasonic exfoliation of h-BN and surface modification of exfoliated BNNSs. Firstly, 0.8 g of h-BN powder was mixed well with 100 mL of IPA, and the intermittent mode of ultrasound was used in the ultrasonic cell comminuter for 6 h. After standing for 24 h to remove the unexfoliated bulk h-BN, the supernatant was collected and then washed with deionized water, followed by drying in a freeze dryer for 12 h to obtain the white and loose powder, which was marked as exfoliated BNNSs. Subsequently, the silane coupling agent KH560 was added into a three-necked flask containing a quantitative amount of water, 2% acetic acid solution and IPA solution, stirring and refluxing for 2 h to create a hydrolysis solution for the silane coupling agent. Then, the prepared BNNSs were added to the composite system, which was heated and refluxed continuously for 3 h. The reflux temperature was set at 60 °C to fabricate m-BNNSs.

### 2.3. Preparation of m-BNNSs/PSAs-Ori Thermally Conductive Composites

**Figure 1:**
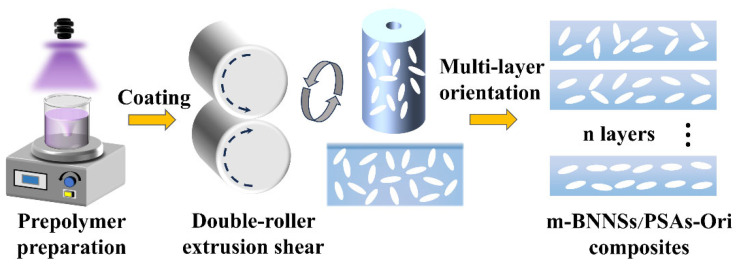
Preparation process of m-BNNSs/PSAs-Ori composites.

Firstly, the three monomers BA, HEA and AA were mixed in a certain ratio, the initiator TPO was added, and the prepolymer with a certain viscosity was formed under UV irradiation after stirring uniformly, and was cooled to room temperature and set aside. Then the initiator TPO and cross-linker HDDA were added to the acrylate prepolymer, stirred for 30 min until well mixed, and then m-BNNS fillers were added with the masses of m-BNNSs of 5, 10, 15, 20 and 25 g (per 100 g of prepolymer), and coated after stirring for 4 h. The m-BNNS fillers were added to the prepolymer with masses of 5, 10, 15, 20 and 25 g (per 100 g of prepolymer). During the coating process, the thickness of the adhesive layer was controlled by adjusting the roller spacing using a C ruler, and the mixture was coated onto a 100 μm thick PET film. After curing, the release film was removed, the second coating was performed, and so on up to the fifth layer, controlling the total adhesive layer thickness of 150 μm, and each component of the pressure-sensitive adhesive was coated with 1–5 layers, respectively, and then cut into standard samples and tested after UV curing. (Figure 1)

#### Experimental Principle

(1) TPO photoinitiated decomposition:

### 2.4. Characterization

The crystal structure of m-BNNSs/PSAs-Ori composites was tested using a X-ray diffractometer (D8 Discover, Ingelfingen, Germany). The morphology and microstructure of the m-BNNSs/PSA-Ori composites were observed using field emission scanning electron microscopy (Nova Nano SEM 450, FEI, Hillsboro, OR, USA). A thermogravimetric analyzer (TGA, SDT Q600, TA Instruments, New Castle, DE, USA) was used to perform heat loss tests on composite samples. Loop tack force and 180° peeling force tests of pressure-sensitive adhesives on stainless steel substrates using a thermostatic tape holding force tester (SM-1600, Dongguan Lize Electronic Technology Co., Ltd., Dongguan, China). The TC of the composites was measured at room temperature by the transient hot-wire method using a TC3000E thermal conductivity meter from Summer Creek Electronic Technology Co., Shenzhen, China and the average of several measurements was obtained. The surface temperature distribution and thermal images of the samples were visualized with a Testo infrared camera (Testo-885-2, Testo, Shanghai, China).

## 3. Results and Discussion

### 3.1. Thermal Conductivity of m-BNNSs/PSAs-Ori Composites

**Figure 2:**
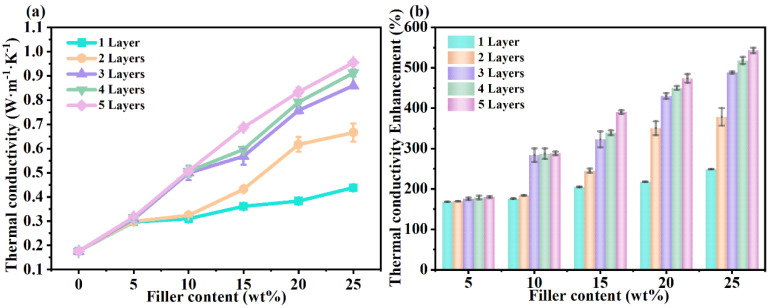
Thermal conductivity of m-BNNSs/PSAs—Ori composites with different number of layers as a function of filler content. (**a**) Variation of thermal conductivity. (**b**) Enhanced thermal conductivity.

Figure 2 shows the TC of pressure-sensitive adhesive composites prepared with different numbers of coats as a function of filler content. As can be seen from Figure 2, the TC increases with increasing filler amount. In contrast, the pressure-sensitive adhesive composites coated with a single layer exhibit the lowest TC, which is ascribed to the random distribution and alignment of m-BNNSs within the matrix. Increasing the number of coating times significantly increased the TC of the composites at coating times less than 3. The TC of the composites coated with three layers at a filler amount of 25 wt% amounted to 0.8347 W/(m·K), which was a 474% increase over the pure PSAs, and that of the pressure-sensitive adhesive coated with five layers reached 0.9552 W/(m·K), which was a 543% increase over the pure PSAs. This is due to the fact that the m-BNNS nanosheets themselves are an anisotropic filler, and further double-roll extrusion shear cycling process makes the m-BNNS nanosheets in the composite fibers well-oriented, which provides a pathway for phonon transport. The highly oriented m-BNNS nanosheets effectively improve the thermal properties of m-BNNSs/PSAs-Ori composite fibers by aligning and interconnecting inside the composite fibers, thus providing a large number of TC channels; as can be seen from the XRD patterns, it is shown that the ordering degree of the composite material is enhanced after multiple coatings with double-roll extrusion, which is conducive to the phonon transmission and reduces scattering.

### 3.2. The SEM Images of m-BNNSs/PSAs-Ori Composites

**Figure 3:**
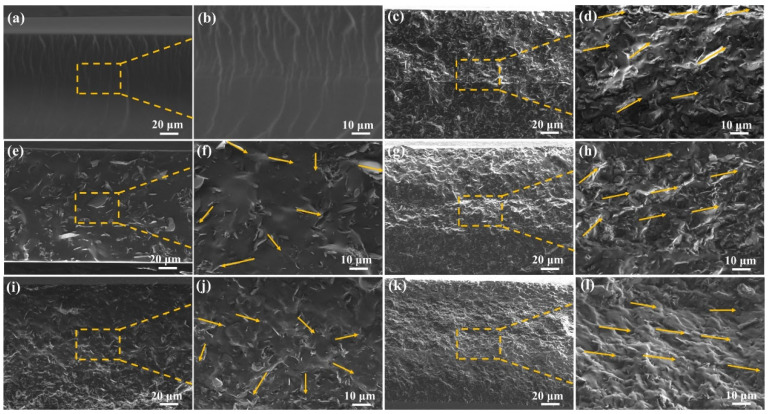
SEM images of (**a**,**b**) pure PSAs, (**e**,**f**) m-BNNSs/PSAs-Ori-1L, (**i**,**j**) m-BNNSs/PSAs-Ori-2L, (**c**,**d**) m-BNNSs/PSAs-Ori-3L, (**g**,**h**) m-BNNSs/PSAs-Ori-4L, (**k**,**l**) m-BNNSs/PSAs-Ori-5L composites. Yellow arrows indicate the orientation of m-BNNSs.

Scanning electron microscopy was used to observe the cross-section morphology of the composites to evaluate the distribution and orientation of m-BNNSs in the pressure-sensitive adhesive matrix after multiple double-roll extrusions. Figure 3 shows cross-sectional SEM images of pure PSAs, m-BNNSs/PSAs-Ori-1L, m-BNNSs/PSAs-Ori-2L, m-BNNSs/PSAs-Ori-3L, m-BNNSs/PSAs-Ori-4L and m-BNNSs/PSAs-Ori-5L composites. Yellow arrows indicate the orientation of m-BNNSs. As shown in Figure 3, the cross-section morphology of the pure PSAs pressure-sensitive adhesive was smooth and uniform, and there was no m-BNNS filler in the matrix. In the m-BNNSs/PSAs-Ori-1L~5L composites, the m-BNNSs were uniformly distributed in the matrix and no agglomeration was found. After one coating, the fillers were arranged randomly in the matrix, and with the increase of the coating times, they tended to be arranged parallel to each other in a “flat” orientation. This is due to the fact that the multilayer extrusion during the double-roller milling process reduces the rotational freedom of the m-BNNSs in the outward direction (perpendicular to the substrate plane) through mechanical constraints, which encourages the nanosheets to further tightly stack in the inward direction (parallel to the substrate plane) and form a two-dimensional network with consistent orientation. Although this structural feature strengthens the continuity of the heat conduction paths of BNNSs in the in-plane direction, the edge contacts of BNNSs at the interlayer interfaces can partially bridge the out-of-plane heat conduction paths of the neighboring layers through the stacking of multilayer coatings. The increase in the degree of orientation observed in the SEM is consistent with the results of the XRD test. Meanwhile, the m-BNNSs still had an excellent orientation state at other filler contents in m-BNNSs/PSAs-Ori-5L, as shown in Figure S1.

### 3.3. Orderliness of m-BNNSs/PSAs-Ori Composites

The ordered arrangement of fillers in the polymer matrix is the key to composite performance enhancement [40]. In order to verify whether multiple coatings are effective for the ordered arrangement in the filler polymer, the degree of ordering of the filler in the polymer matrix was characterized by X-ray diffractometry. In each crystal plane of h-BN, 26.7°, 41.5° and 55.1° correspond to (002), (100) and (004) peaks, respectively, where (002) and (100) peaks indicate that the lamellae are in a horizontal or vertical state, respectively. The degree of ordering α is introduced here, which is calculated as follows:

$$\alpha =I_{002}/I_{100}$$

**Figure 4:**
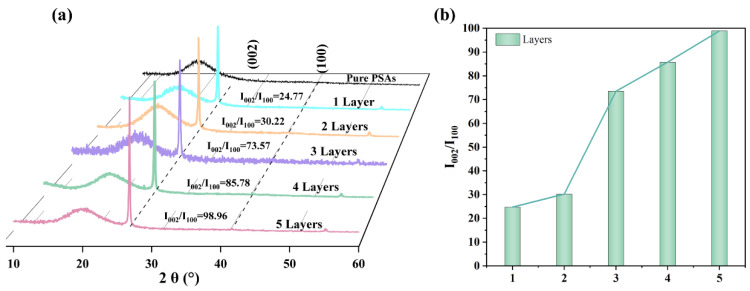
(**a**) XRD curves of pressure-sensitive adhesives produced with different number of coats as a function of filler content. (**b**) Orientation of pressure-sensitive adhesive composites produced with different number of coats (I_002_/I_100_).

By α, the lamellar orientation of m-BNNSs in the composites can be explained from a certain perspective, which determines the formation of the final TC pathway. Figure 4 shows the XRD of the pressure-sensitive adhesive composites prepared with different number of coating times and their corresponding ordering degree histograms. As shown in Figure 4a,b, the α of the m-BNNSs/PSAs-Ori composites are 24.77, 30.22, 73.57, 85.78 and 98.96 in order with the increase of coating times, indicating that the increase of coating times facilitates the ordering of the fillers in the polymer matrix. At the same filler amount, increasing the number of coats leads to an increase in α, which can be attributed to the following factors: (1) m-BNNSs, due to their large aspect ratio, significantly increase their contact area with the PSAs substrate, which reduces the appearance of voids and improves the filler’s orientation in the polymer during double-roll extrusion; (2) at a fixed adhesive layer thickness, the multilayer coating further restricts the m-BNNSs’ rotation from in-plane to out-of-plane, so that the m-BNNSs are more tightly stacked in the in-plane under the double-roll extrusion, thus realizing the high degree of orientation of m-BNNSs in the matrix.

### 3.4. Infrared Spectra of m-BNNSs/PSAs-Ori Composites

**Figure 5:**
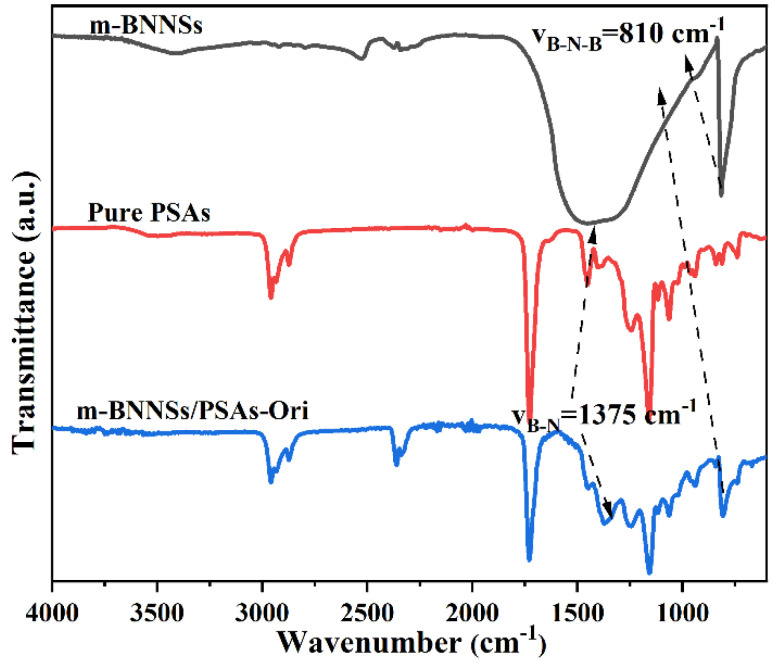
IR spectra of m-BNNSs, pure PSAs and m-BNNSs/PSAs—Ori composites.

The m-BNNSs, pure PSAs and m-BNNSs/PSAs-Ori composites were further compared by IR spectra as shown in Figure 5. Compared with the pure pressure-sensitive adhesive without fillers, the IR spectra of m-BNNSs/PSAs-Ori composites showed new peaks at 1375 cm^−1^ and 810 cm^−1^, which were attributed to the B-N in-plane stretching vibration peak of BN and the B-N-B out-of-plane bending vibration peak, respectively. In the infrared profiles of m-BNNSs/PSAs-Ori composites, the characteristic peaks are similar to those of pure PSAs except for the interference of CO_2_ (2300 cm^−1^), which indicates that the pressure-sensitive adhesive was successfully prepared.

### 3.5. Viscoelasticity of m-BNNSs/PSAs-Ori Composites

**Figure 6:**
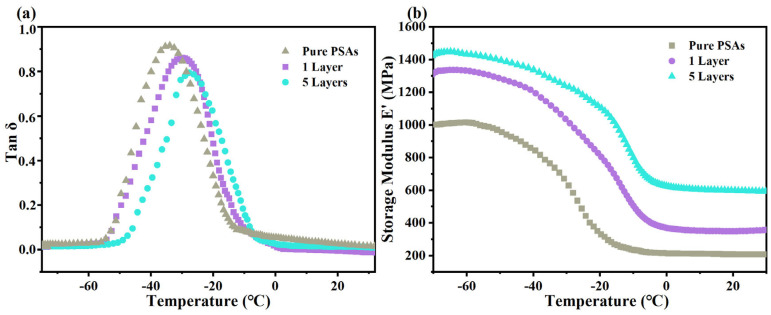
Coated single layer of pure PSAs, single and five layers of m—BNNSs/PSAs—Ori pressure—sensitive adhesive with the same filler amount (**a**) tan δ—T, (**b**) E′—T.

The energy dissipation of a material under cyclic loading is called damping [41]. The damping factor tan δ can be utilized to assess the potential of a material to dissipate and absorb energy. The tan δ of the composites measured over a range of temperatures is shown in Figure 6a. The addition of fillers decreases the tan δ of the composites and the tan δ of the composites coated with five layers of pressure-sensitive adhesive is the lowest. A previous study found that fillers act as barriers in composites, restricting the movement of polymer chains. This diminishes the flexibility, molecular motion and consequently the damping factor of the composite [42]. Furthermore, the damping characteristics of heterogeneous systems are influenced not solely by interfacial bonding, but also by additional factors such as interfacial thickness, fiber flexure, fiber fracture, matrix crazing and the generation of voids resulting from fiber pull-out [43]. In this study, pure PSAs showed maximum mobility with the highest damping factor. After adding fillers, the composites coated with five layers have a lower damping factor due to the stronger bonding of fillers to the matrix and the reduced molecular mobility of the polymer obviously leads to a lower damping factor, on the other hand adding fillers increases the stiffness of the composites and the degrees of freedom of the polymer chains are restricted, which limits the molecular mobility thus lowering the damping factor of the composites.

The glass transition temperature (T_g_) is the lowest temperature at which a molecular chain segment can move, and its level is directly related to the flexibility of the molecular chain. In polymer composites, the T_g_ value can be determined by the peak value of the damping factor. As shown in Figure 6a and Table 1, the T_g_ of pure PSAs is −34 °C, and with the addition of 25 wt% filler, the T_g_ of the composites coated with 1-layer increases to −30 °C, and the value of T_g_ of the composites coated with five layers increases to −28 °C. This shift in T_g_ to higher temperatures may be related to the specific decrease in mobility, and this alteration may stem from the variation in the crosslink density of the network.

The energy storage modulus (E′) serves to measure a material’s capacity to store applied energy [44]. Figure 6b shows the effect of temperature on the energy storage modulus of the composites. Based on the curves, it can be seen that for each specimen, the energy storage modulus decreases with increasing temperature, and this decrease in the energy storage modulus represents the phase transition from the glassy to the rubbery state in polymer composites, which usually occurs after the T_g_ [45]. The constituents of a composite in the glassy state are closely packed and demonstrate robust intermolecular forces, which contribute to elevated energy storage moduli [46,47,48]. As the temperature rises, molecular motion intensifies, causing molecular chains to relax. Consequently, this leads to a reduction in stiffness and thus a lower value of the energy storage modulus. The coated five-layer pressure-sensitive adhesive composites exhibit the highest energy storage modulus. This property is influenced by the extent of interfacial bonding between the filler and the matrix. Additionally, the energy storage modulus can be enhanced by the stiffening effect that arises from the interaction between the filler and the matrix, thereby facilitating effective stress transfer [49].

### 3.6. Thermal Stability Properties of m-BNNSs/PSAs-Ori Composites

**Figure 7:**
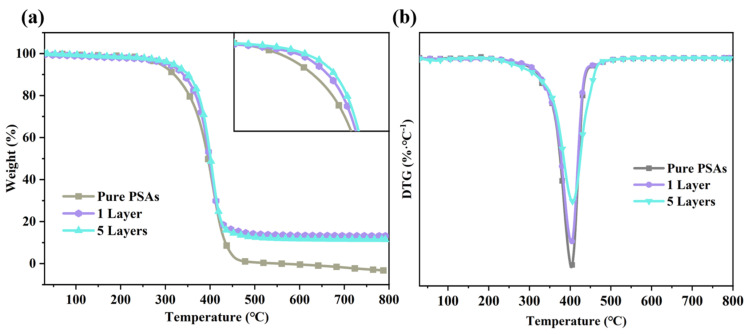
(**a**) TG curves and (**b**) DTG curves of coated single—layer pure PSAs, single—layer and five—layer m-BNNSs/PSAs—Ori pressure—sensitive adhesives.

The stability of thermal management materials under high-temperature conditions is a prerequisite for their thermal function. The thermal stability of composites coated with different numbers of times of pressure-sensitive adhesive was evaluated by TGA. In the thermogravimetric analysis of polymers, the onset of decomposition temperature is an important indication of thermal stability, but since the onset of decomposition temperature is difficult to measure, it was replaced by using the temperature T_10_ at 10% mass loss and T_40_ at 40% mass loss, which is recorded in Table 2. Figure 7 shows the heat weight loss curves of m-BNNSs/PSAs-Ori composites coated once and five times with one layer of pure PSAs and the same amount of filler, respectively, in the range of room temperature to 800 °C. It can be seen that the T_10_ of one layer of pure PSAs is 320 °C, the T_10_ of m-BNNSs/PSAs-Ori composites with the addition of 10 wt% of m-BNNSs/PSAs-Ori composites increase to 340 °C, and the T_10_ of the composites made after five coats increases to 352 °C, and the amount of residual C improves, and similarly the T_40_ appears to be increased to different degrees. It shows that the m-BNNSs have a certain strength and improve the thermal stability of m-BNNSs/PSAs-Ori composites [37]. The maximum weight loss was observed in the range of 300–400 °C. Several curves had similar thermal degradation behavior, which is due to the similar chemical structure of the polymers. In addition, by increasing the number of coatings, the onset decomposition temperature can be increased and the thermal stability of the composites can be improved.

### 3.7. Thermal Diffusion Ability

**Figure 8:**
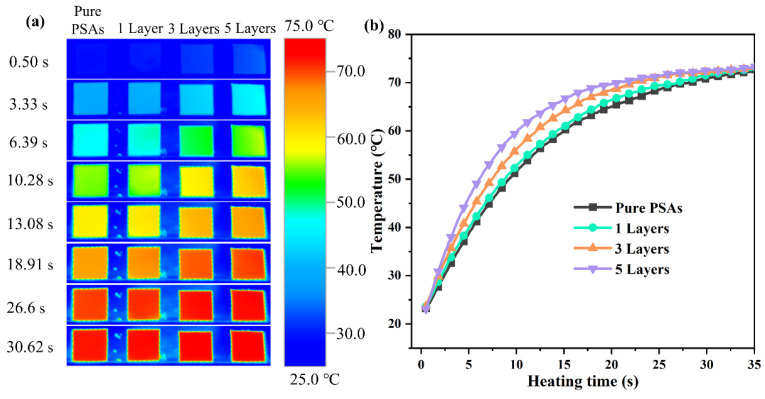
(**a**) Infrared thermograms of composites prepared with different number of coating times during the heating process, (**b**) surface temperature variation of composites prepared with different number of coating times.

In order to visualize the heat transfer efficiency of the composites, an infrared camera was used to record the difference in temperature response during heating, as shown in Figure 8. Compared with the single-layer coating, the surface temperature of the composites coated with five layers rises rapidly, and the surface temperature has reached 60 °C after 10 s of heating. And, according to the infrared thermography result, it can be seen that the color change per unit time is more obvious in the heating process of the composite material coated with five layers, which indicates that its heat conduction is remarkable, can effectively transfer heat, and has a good potential in the field of thermal management. And, according to Figure S2, it can be seen that the thermal responsiveness of the m-BNNSs/PSAs-Ori-5L composites becomes stronger with the increase of the filler content, and these results further confirm the importance of the number of multilayer stacking coatings for enhancing the thermal conductivity. More importantly, this result is consistent with the previous discussion.

**Figure 9:**
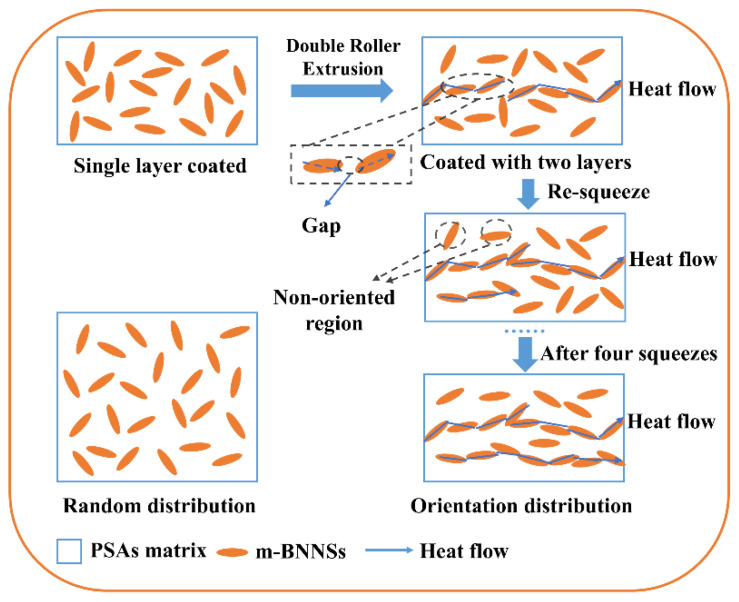
Schematic representation of the thermal conduction mechanism of m-BNNSs/PSAs-Ori and m-BNNSs/PSAs composites.

A schematic of the effective heat flow network in the m-BNNSs/PSAs-Ori composites is given in Figure 9, indicating that the composites with better arrangement of m-BNNS sheets have higher thermal conductivity. In addition, the m-BNNSs were randomly dispersed within the matrix prior to compression; however, they exhibited a tendency to align in the in-plane direction following five compression cycles. Therefore, compression is regarded as a crucial factor in realizing the orientation and stacking of the m-BNNS sheets [5], which facilitates the overlapping of fillers, which leads to the formation of thermal conduction paths. As a result, the m-BNNSs/PSAs-Ori composites demonstrate superior thermal conductivity in comparison to the m-BNNSs/PSAs composites.

### 3.8. Mechanical Properties of m-BNNSs/PSAs-Ori Composites

As a thermal interface material, pressure-sensitive adhesive, in addition to ensuring a high thermal conductivity, excellent pressure-sensitive properties are also indispensable. This pressure-sensitive property ensures that the adhesive will adhere well to the surface under different pressure conditions and fit tightly, thus realizing the ideal heat transfer effect. Whether in light pressure or large pressure application scenarios, it can accurately meet the pressure sensitivity requirements to build a reliable bridge for the transfer of heat between different components, so that the thermal interface materials can be stable in complex and changing working conditions to play its heat conduction function.

**Figure 10:**
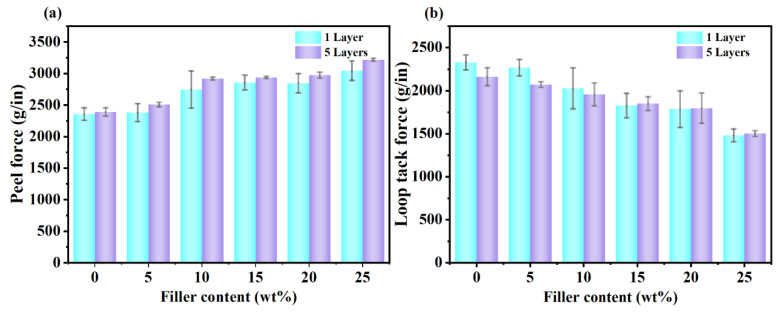
Pressure-sensitive properties of m-BNNSs/PSAs-Ori-1L and m-BNNSs/PSAs-Ori-5L composites (**a**) 180° peeling force, (**b**) loop tack force.

In order to compare the effect of the number of coats on the pressure-sensitive properties, the m-BNNSs/PSAs-Ori-1L and m-BNNSs/PSAs-Ori-5L composites were tested for 180° peeling force and loop tack force, respectively (Figure 10). It can be seen from Figure 10. that with the increase in the number of coating times, the peeling force is improved, which is due to the increase in the number of effective bonding layers formed by the pressure-sensitive adhesive on the surface of the adhesive, the interaction between the adhesive layers is enhanced, and the cohesive force is improved [50]. At the same time, more pressure-sensitive adhesive molecules contact and interact with the surface of the adhesive, forming more bonding points, making the 180° peeling force gradually increase. The addition of fillers inevitably reduces the initial tack of the material. This is because the addition of fillers changes the original structure and properties of the material. Filler particles tend to occupy a certain amount of space, making the distribution of effective bonding components in the pressure-sensitive adhesive system relatively sparse, reducing the number of points of action that can form an effective bond when initially contacting the surface of the object to adhere to.

### 3.9. Electrical Insulation Properties of m-BNNSs/PSAs-Ori Composites

**Figure 11:**
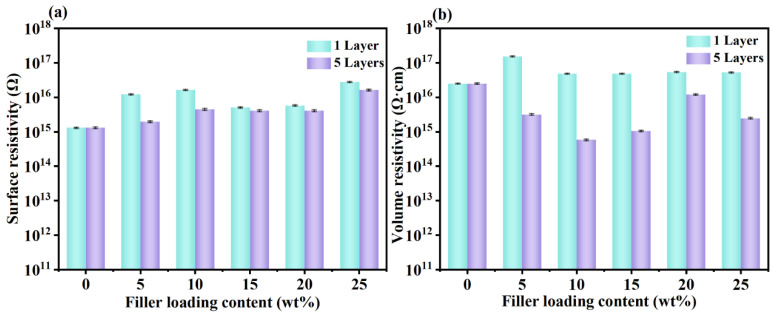
Plots of surface resistance (**a**) and volume resistance (**b**) of m-BNNSs/PSAs-Ori-1L and m-BNNSs/PSAs-Ori-5L composites with filler content.

Good insulating properties are an important guarantee for the practical application of thermally conductive adhesives as encapsulation or heat dissipation materials in high-density integrated systems. In order to investigate the insulating properties of the composites, the surface resistivity and volume resistivity of the composites with different number of coating times and different filler filling amounts were determined using a ZC36 high resistivity meter, and as shown in Figure 11, the surface resistivity and volume resistivity of the pure PSAs were 1.31 × 10^15^ Ω and 2.5 × 10^16^ Ω·cm, respectively. Compared with the single-layer pressure-sensitive adhesive, the surface resistance and volume resistivity of the pressure-sensitive adhesive prepared by five coats showed different degrees of decrease, in which the surface resistivity did not differ much, and the volume resistivity trend was more obvious, and the lowest value appeared in the filler content of 10 wt%, and the volume resistivity was 6.72 × 10^14^ Ω·cm (>10^9^ Ω cm), which was slightly lower than that of the pure PSAs. It has been demonstrated that the volume resistivity can meet the demand for internal diaphragm materials for miniaturized electronic devices when the materials have a volume resistivity greater than 10^9^ Ω cm [51,52]. Therefore, the composites have excellent thermal conductivity and electrical insulation properties.

## 4. Conclusions

In conclusion, polyacrylate PSAs were successfully prepared by an environmentally friendly and efficient UV-bulk polymerization method, combined with a simple calendering process to achieve the preparation of multilayer coated m-BNNSs/PSAs-Ori composites. The highly oriented and flexible m-BNNSs/PSAs-Ori composites exhibit excellent thermal diffusion ability, electrical insulation and mechanical properties. By comparing the TC with that of pure PSAs as well as monolayer m-BNNSs/PSAs composites, the m-BNNSs/PSAs-Ori composites showed excellent thermal conductivity. At a m-BNNS mass fraction of 25%, the thermal conductivity of the composites coated with three layers reached 0.8347 W/(m·K), which was a 474% increase over pure PSAs, and the TC of the PSAs coated with five layers reached 0.9552 W/(m·K), which was a 543% increase over pure PSAs. This work provides a reasonable design scheme for polyacrylate PSA composites, and then promotes their practical application in thermal management.

## Figures and Tables

**Table 1:** Summary of viscoelastic parameters of composites prepared by coating different number of layers with pure PSAs, 25 wt% filler amounts.

Composite	T_g_ Obtained from Tan δ Curves (°C)	E′ (MPa) at 25 °C
Pure PSAs	−34	214
1 Layer m-BNNSs/PSAs	−30	358
5 Layers m-BNNSs/PSAs	−28	604

**Table 2:** Thermal decomposition data of m-BNNSs/PSAs-Ori composites.

Sample	T_10_/°C	T_40_/°C	T_max_/°C	Residue/%
Pure PSAs	320	385	403	1.03
1 Layer	340	391	403	14.18
5 Layers	352	394	405	13.35

## Data Availability

Data will be made available on request.

## Supplementary Materials

The following supporting information can be downloaded at: https://www.mdpi.com/article/10.3390/polym17121604/s1, Figure S1. SEM images of m-BNNSs/PSAs-Ori-5L composites (a) pure PSAs, (b) m-BNNSs/PSAs-Ori-5L-5wt%, (c) m-BNNSs/PSAs-Ori-5L-10wt%, (d) m-BNNSs/PSAs-Ori-5L-15wt%, (e) m-BNNSs/PSAs-Ori-5L-20wt% and (f) m-BNNSs/PSAs-Ori-5L-25wt%; Figure S2. (a) Infrared thermograms of m-BNNSs/PSAs-Ori-5L composites with different filler amounts during the heating process, (b) Surface temperature variation of m-BNNSs/PSAs-Ori-5L composites with different filler amounts.
