# Supplementary material for: Controllable Preparation of Oriented Boron Nitride Nanosheets/Polyacrylate Pressure-Sensitive Adhesive Composites with Enhanced Thermal Conductivity
Source: Polymers (Basel). 2025 Jun 9;17(12):1604. doi: 10.3390/polym17121604 (PMC12197259; doi:10.3390/polym17121604)
Supplement: Supplementary file 1 [file polymers-17-01604-s001.zip › polymers-3622191-supplementary.pdf]

Supplementary Information

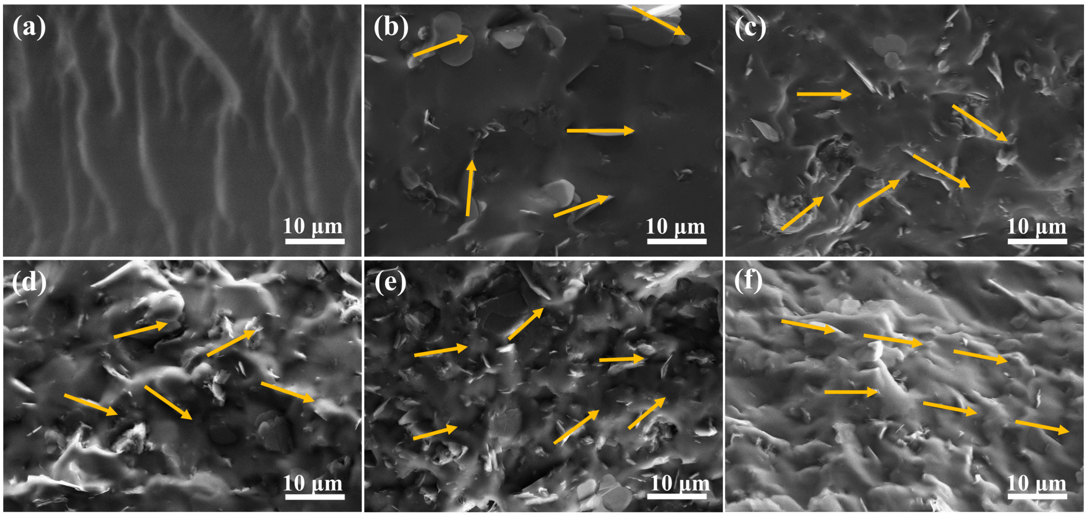

Figure S1 SEM images of m-BNNSs/PSAs-Ori-5L composites (a) pure PSAs, (b) m-BNNSs/PSAs-Ori-5L-5wt%, (c) m-BNNSs/PSAs-Ori-5L-10wt%, (d) m-BNNSs/PSAs-Ori-5L-15wt%, (e) m-BNNSs/PSAs-Ori-5L-20wt% and (f) m-BNNSs/PSAs-Ori-5L-25wt%.

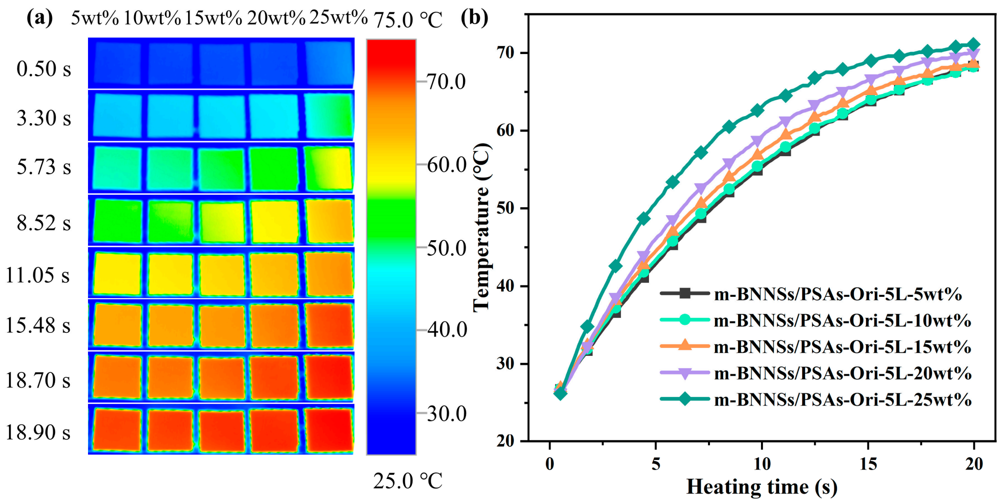

Figure S2 (a) Infrared thermograms of m-BNNSs/PSAs-Ori-5L composites with different filler amounts during the heating process, (b) Surface temperature variation of m-BNNSs/PSAs-Ori-5L composites with different filler amounts.
